# Exosomal MiRNA Transfer between Retinal Microglia and RPE

**DOI:** 10.3390/ijms21103541

**Published:** 2020-05-17

**Authors:** Dorothea R. Morris, Sarah E. Bounds, Huanhuan Liu, Wei-Qun Ding, Yan Chen, Yin Liu, Jiyang Cai

**Affiliations:** 1 Department of Ophthalmology & Visual Sciences, University of Texas Medical Branch, Galveston, TX 77555, USA; dormorri@utmb.edu (D.R.M.); Yan-Chen@ouhsc.edu (Y.C.); 2 Department of Physiology, University of Oklahoma Health Sciences Center, Oklahoma City, OK 73104, USA; Sarah-Bounds@ouhsc.edu (S.E.B.); Huanhuan-Liu@ouhsc.edu (H.L.); 3 Department of Pathology, University of Oklahoma Health Sciences Center, Oklahoma City, OK 73104, USA; WeiQun-Ding@ouhsc.edu; 4 Dean McGee Eye Institute, University of Oklahoma Health Sciences Center, Oklahoma City, OK 73104, USA; 5 Department of Neurobiology and Anatomy, University of Texas Health Science Center at Houston, Houston, TX 77030, USA

**Keywords:** RPE, exosome, aging, microglia, inflammation

## Abstract

The retinal pigment epithelium (RPE), the outermost layer of the retina, provides essential support to both the neural retina and choroid. Additionally, the RPE is highly active in modulating functions of immune cells such as microglia, which migrate to the subretinal compartment during aging and age-related degeneration. Recently, studies have highlighted the important roles of microRNA (miRNA) in the coordination of general tissue maintenance as well as in chronic inflammatory conditions. In this study, we analyzed the miRNA profiles in extracellular vesicles (EVs) released by the RPE, and identified and validated miRNA species whose expression levels showed age-dependent changes in the EVs. Using co-culture of RPE and retinal microglia, we further demonstrated that miR-21 was transferred between the two types of cells, and the increased miR-21 in microglia influenced the expression of genes downstream of the p53 pathway. These findings suggest that exosome-mediated miRNA transfer is a signaling mechanism that contributes to the regulation of microglia function in the aging retina.

## 1. Introduction

Postmitotic cells, such as neurons and the retinal pigment epithelium (RPE), tend to accumulate macromolecular damage and degenerate early in the aging process [1,2]. In addition to the intrinsic mechanisms of these cells, in neuronal tissue like the retina, the rate of aging and the progression of degeneration are strongly influenced by the surrounding microenvironment and cell–cell interactions. Inflammation and immune responses, which arise from interactions between stressed postmitotic cells and immune cells of the retina, can act as accelerating factors of aging and age-related retinal diseases [3,4]. As transferrable genetic materials between different cell types, microRNAs (miRNAs) play important roles in modulating inflammation associated with organism longevity and tissue aging [5,6,7].

Initially identified in *Caenorhabditis elegans*, miRNAs have been implicated in regulating the expression of about 60% of mammalian messenger RNA (mRNA) species [8,9]. Mature miRNAs are single-stranded RNAs of about 22 nucleotides that bind to target mRNAs and trigger the mRNA degradation and/or protein translational inhibition [10,11]. Age-related changes of miRNA profiles have been reported in various tissues and organs [12,13,14,15], and miRNAs can strongly influence components of major signaling pathways related to aging [16]. In ocular tissues, miRNAs are involved in both physiological processes, such as light/dark adaptation [17] and circadian rhythm [18], and many disease processes [19,20,21,22,23], including age-related macular degeneration (AMD) [24]. It is found that miRNAs exist in exosomes, and exosomal miRNAs can be delivered to recipient cells as signaling molecules [25,26,27].

Retinal microglia are residential phagocytes that normally perform surveillance functions and maintain neuronal homeostasis [28]. Parenchymal microglia migrate into the subretinal space during aging and stress conditions, and they facilitate the removal of subretinal debris derived from photoreceptor neurons [29]. In retinal diseases with chronic and low-grade inflammation, such as AMD, aberrant microglia activation can cause neuronal cell damage [29]. The chemotactic signals and activation mechanisms of these microglia remain elusive.

In the current study, we examined the roles of RPE-derived exosomal miRNAs in modulating microglial function. We found that aged RPE cells increased the release of a selective set of miRNAs in their exosomes. Using miR-21 as a proof-of-concept approach, we demonstrated that miR-21 can be transferred between RPE and microglia, and the elevated miR-21 content in microglia affected gene expression in the p53 pathway. The findings suggest that exosome-mediated miRNA transfer can be a signaling mechanism related to chronic inflammation during RPE aging.

## 2. Results

### 2.1. Exosomal miRNA Profiles Differ between Young and Aged RPE

**Figure 1:**
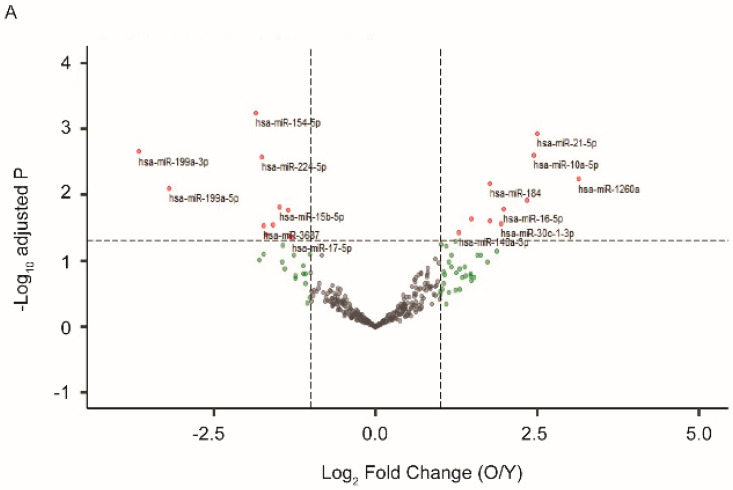

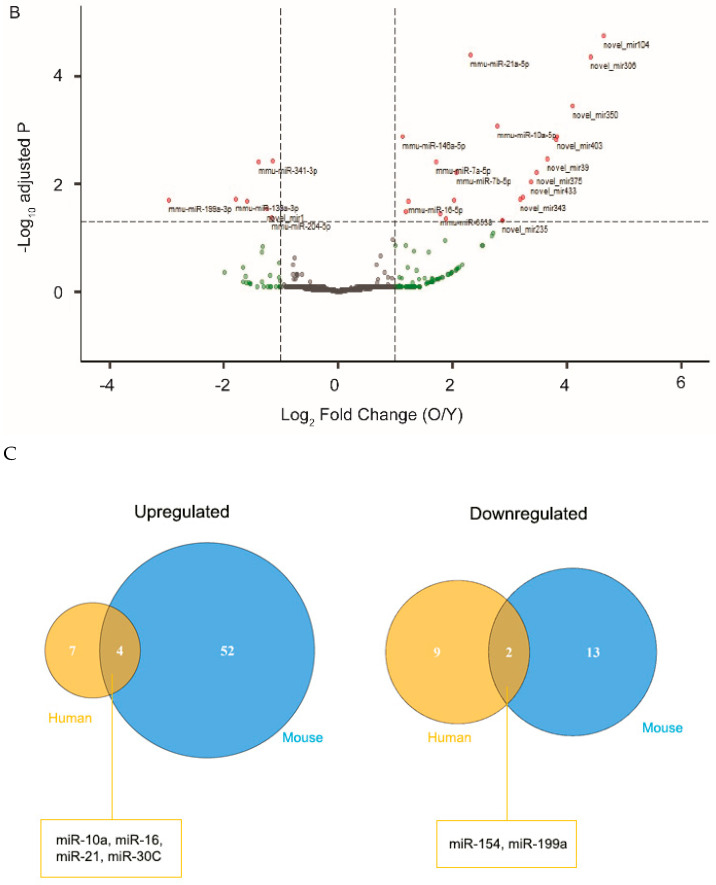
Differential expression of miRNAs in extracellular vesicles (EVs) from young and old retinal pigment epithelium (RPE). EVs were isolated from either cultured human RPE cells (**A**) or mouse RPE/choroid-conditioned medium (**B**). EV-associated RNA was extracted and sequenced. Differential gene expression data were presented as volcano plots to show the fold of change (*x*-axis) and adjusted *p* value (*y*-axis). The horizontal dashed line indicates an adjusted *p*-value < 0.05 threshold, and vertical dashed lines indicate a fold change > 2 threshold. MiRNA species showing significant changes in EVs derived from aged RPE were marked with red and annotated on the plots. (**C**) Venn diagrams illustrating the number of exosomal miRNAs with significantly altered expression between young and aged samples from human or mouse RPEs. Mature sequences of the miRNA species at the intersections are conserved between mouse and human.

We previously established a model of in vitro postmitotic aging using cultured human RPE cells [30]. In media with a defined nutrient composition, confluent RPE cells gradually developed a battery of aging markers and displayed functional loss after four to six weeks in culture [30]. At one and four weeks after the beginning of the postmitotic aging, we isolated EVs that had been released from the cultured RPE cells and compared their miRNA profiles. As shown in Figure 1A, among the 340 miRNA species detected in EVs from RPE, 66 had at least 2-fold up- or down-regulation. Eleven of them, including miR-184, 10a, and 21, were significantly upregulated (Benjamini–Hochberg adjusted *p*-value < 0.05). Eleven of them, including miR-154, 199a, and 224, were downregulated (adjusted *p*-value < 0.05) in EVs from aged RPE.

As an independent validation of the findings from cultured human RPE cells, we next examined miRNA profiles of EVs isolated from mouse posterior eye segments, following ex vivo organ culture. Mice at the age of 4–6 months and 18–22 months were used as young and aged groups, respectively. Similar to the findings from cultured RPE cells, exosomal miRNAs released by the RPE/choroid/sclera explant from old mice had varying profiles as compared to those of young mice (Figure 1B). As seen with RPE culture, several miRNA species, including miRNA-21, were found to have significantly altered levels in EVs derived from aged ex vivo explant (Figure 1C).

**Figure 2:**
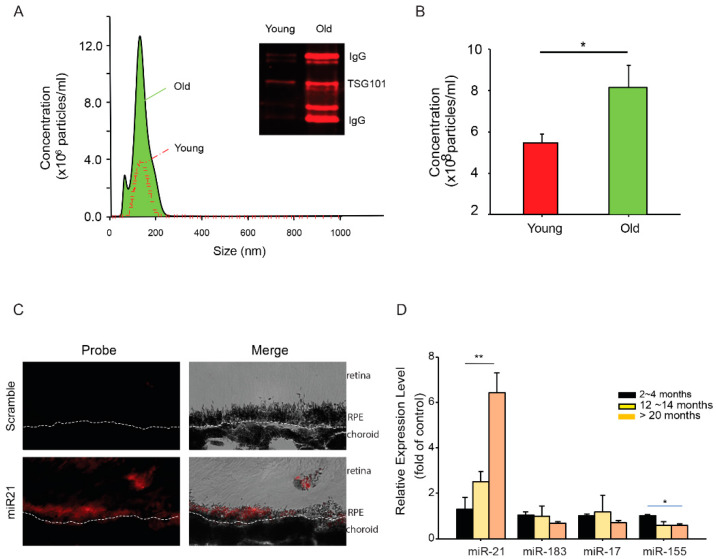
Age-dependent changes in RPE exosomes and miRNAs. (**A**) The size distribution of EVs in culture medium conditioned by young or aged mouse RPE/choroid tissue. Insert: Western blot of exosome marker proteins in isolated EVs. (**B**) Concentration of EV particles in mouse RPE/choroid-conditioned medium, as measured by nanoparticle tracking analyses. (**C**) In situ hybridization of miRNA-21. Panels of fluorescent and bright-field images were presented to show the expression of miR-21 in the RPE. Dashed line indicates the basal side of the RPE. (**D**) Quantitative RT-PCR analyses of selected miRNAs in RPE from mice at different age groups. Data presented are averages of 4 independent experiments (mean ± sem; * *p* < 0.05; ** *p* < 0.01).

To obtain further understanding of the differences of miRNAs in the EVs derived from young and old RPE, we analyzed the size and abundance of EVs released from posterior eyecups using nanoparticle tracking analyses. The size distributions were comparable between EVs from young and aged RPE (Figure 2A). Aged RPE/choroid tissue had about a 2-fold increase (2.2 ± 0.4, mean ± SD) in the amount of EVs released into the medium (Figure 2B). EVs isolated from the eyecups had exosomal marker proteins such as TSG101 (Figure 2A inset), validating the presence of exosomes in our preparation [31]. EVs from aged RPE tissues consistently showed the presence of immunoglobulin (IgG) heavy and light chains (Figure 2A). Similar findings have been recently reported from EVs isolated from diabetic plasma samples [32].

MiR-21 has been implicated in models of retinal diseases such as diabetic retinopathy [33] and oxygen-induced retinopathy [34]. As a proof-of-concept, we selected miR-21 for our functional studies. Using the in situ hybridization approach, we confirmed the expression of miR-21 in the RPE (Figure 2C). When measured for age-dependent changes in the cellular miRNA pool, miR-21 showed an approximate 6-fold increase (6.4 ± 2.3, mean ± SD) in RPE isolated from mice between 20 and 24 months of age, as compared to young (2 to 4 months) and mid-aged (12 to 14 months) mice (Figure 2D). The aged-dependent increase was not universal for all miRNA species, as the levels of many other miRNAs (e.g., miR-183 and miR-17) remained comparable in the aged RPE. Thus, our data demonstrated a selective increase of miR-21 in both cellular and extracellular compartments of the aged RPE.

### 2.2. Transfer of miR-21 between RPE and Retinal Microglia

**Figure 3:**
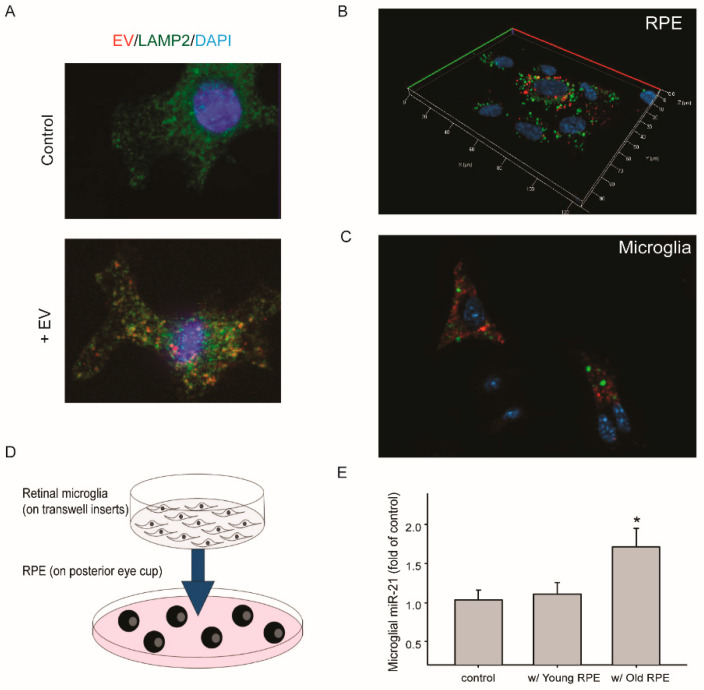
Transferred miR-21 between cultured RPE and microglia. (**A**) Microglial uptake of purified EVs from RPE. EVs were labeled with the lipophilic dye DiI (red). Immunostaining of LAMP2 was performed (green), along with DAPI staining of nuclei. (**B**) and (**C**) miR-21 transferred via RPE-conditioned medium. (**B**) Fluorescein-labeled miR-21 mimics (green) were transfected into cultured mouse RPE cells. (**C**) One day after transfection, the conditioned medium was collected and used to treat cultured microglia. Red: LysoTracker Red. (**D**) Schematic model of RPE and microglia co-culture. The posterior eyecups containing RPE/choroid tissues were ex vivo cultured on 6-well plate, and microglia were grown on transwell insert. (**E**) Quantitative RT-PCR analyses of miR-21 levels in microglia after being co-cultured with RPE/choroid from either young or aged mice, compared to that of the control (without RPE co-culture). Data presented are the average of 3 independent experiments (mean ± sem; * *p* < 0.05).

With aging, retinal microglia migrate from the inner layer to the sub-retinal space [35,36]. Its proximity to the RPE may promote the uptake of EVs released by aged RPE. To test this hypothesis, we examined the transfer of EVs and their miRNAs between RPE and retinal microglia. When cultured microglia were treated with RPE-derived EVs, the uptake and intracellular presence of EVs were detected after 30 min (Figure 3A), and some of the EVs showed co-localization with lysosome marker protein LAMP2. Two methods were utilized to measure the transfer of miR-21 between RPE and retinal microglia (Figure 3B,C). First, fluorescein-conjugated miRNA-21 was transfected into cultured mouse RPE cells (Figure 3B). One day after transfection, RPE-conditioned medium was used to treat microglia that had been grown on coverslips. After two hours, the transfer of miR-21 was detected by fluorescence microscopy (Figure 3C). In the second approach, retinal microglia grown on transwell inserts were co-cultured with eyecups prepared from mice at different ages (Figure 3D). The basal level of miR-21 in microglia was low. Co-culture with RPE/choroid tissues from aged mice (18 to 22 months), but not that from young mice (4 to 6 months), upregulated miR-21 in microglia (Figure 3E).

**Figure 4:**
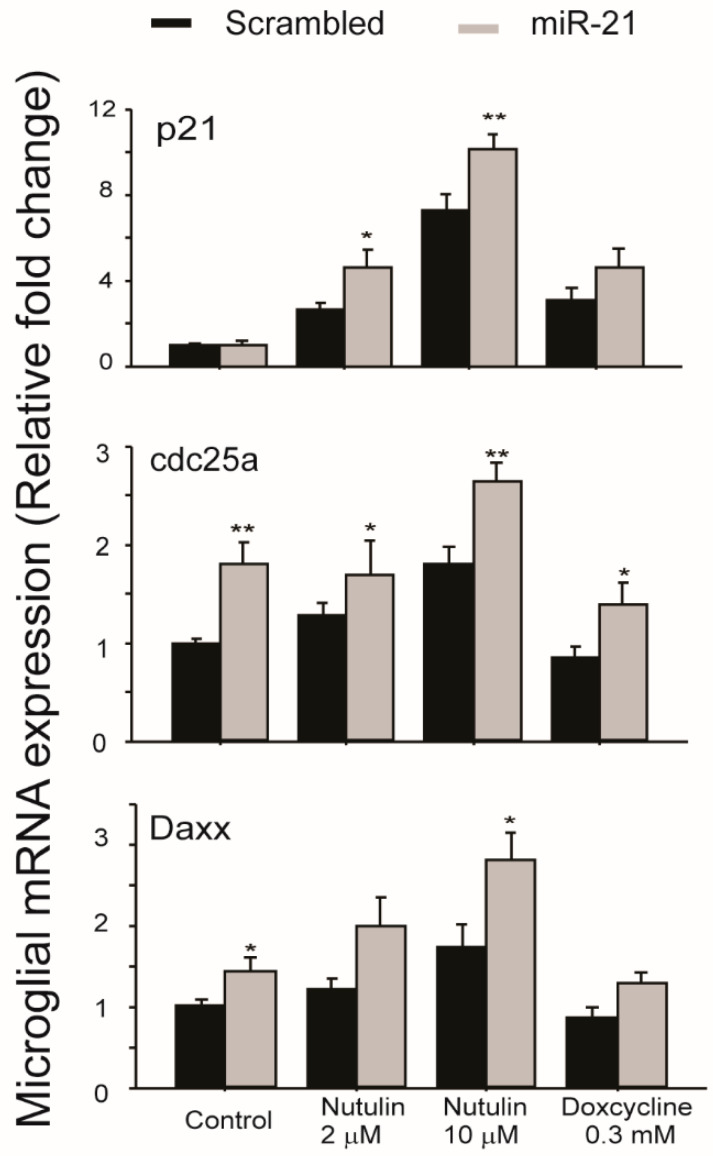
Microglial gene expression after transfection with miR-21 mimics. Cells were transfected with either miR-21 mimics or control scrambled miRNA. At 2 days after transfection, cells were treated with either nutulin or doxycycline at the indicated concentrations for 16 h. Quantitative RT-PCR analyses were performed to measure the relative levels of gene expression downstream of the p53 pathway. Data presented are the average of 4 independent experiments (mean ± sem; * *p* < 0.05; ** *p* < 0.01).

In order to examine the functional consequences of miR-21 transfer between aged RPE and microglia, we used miR-21 mimics to transfect cultured retinal microglia and then examined the expression levels of genes associated with major signaling pathways involved in cell survival, signaling, and stress responses. The results (Figure 4) demonstrated that genes downstream of p53, including *p21*, *Cdc25a*, and *Daxx*, were upregulated in microglia transfected with miR-21 mimics. The basal levels of *Cdc25a* and *Daxx* were increased by miR-21 mimics. After exposure to p53 activating compounds nutulin or doxycycline, the upregulations of all three genes were further potentiated by miR-21 mimics. These data suggested that miR-21, which can be transferred via RPE exosomes, had indirect effects on the p53 pathway in retinal microglia.

## 3. Discussion

Microglia priming and activation are commonly observed during the aging of the central nervous system [37]. Microglia are heterogeneous. Their functions are determined by tissue microenvironments, anatomical locations, and interactions with other cell types. In the aging retina, microglia migrate to the subretinal space, and their interactions with the RPE can be a crucial factor in determining whether they are neuroprotective or neuroinflammatory [35]. The underlying mechanisms are under investigation. Findings from our current study suggest that the transferring of miRNAs via RPE-derived exosomes can be a regulatory mechanism that modulates the functional status of the subretinal microglia.

Using miR-21 as a proof-of-concept, we demonstrated that an enrichment of miR-21 in exosomes was released from aged RPE. Additionally, we showed that miR-21 can be transferred to microglia under co-culture conditions. The endogenous level of miR-21 was low in microglia, and the increase in miR-21 content due to exosome-mediated transfer influenced the gene expression related to the p53 pathway. Moderate but significant increases in *p21*, *Daxx*, and *Cdc25a* expressions were detected both at basal level and after exposure to compounds that activated p53. The upregulation of p53-regulated genes by miR-21 has been reported in cancer cells [38], but not in the context of exosome transfer. The effects of miR-21 in the retina are not limited to microglia. A number of previously published papers report that miR-21 can modulate retinal endothelial cell function by targeting specific signaling proteins such as peroxisome proliferator-activated receptor-α [33] or tissue inhibitor of matrix metalloproteinases-3 [34].

The increased release of miR-21 and other miRNAs in RPE exosomes was likely due to multiple mechanisms. With nanoparticle tracking analyses and Western blot analyses, we found that aged RPE had increased release of exosomes and other EVs. The intracellular miR-21 level was increased in aged RPE, which might contribute to its increase in exosomes. On the other hand, recent literature suggests that the sorting and enrichment of certain miRNA species in exosomes are mediated by their binding proteins [39,40]. Similar mechanisms can be explored in the RPE in future studies. A technical limitation of our study was the use of multiple methods for EV isolation. Extraction of EV RNA was based on column trapping and purification. For Western blot analyses, EVs were precipitated with polymer. Samples measured by NanoSight were not enriched or purified. We acknowledge that the EV populations analyzed in these different experiments may not be equivalent.

The amount of transfected miR-21 mimics was likely to have been much higher than that which microglia will receive from EVs of the RPE. EVs also contain miRNA species other than miR-21 whose expression levels can be influenced by RPE aging. A single miRNA can target hundreds of different mRNAs, and multiple miRNAs often work cooperatively targeting the same genes. Therefore, understanding miRNA regulatory effects will require a detailed description of active miRNA regulatory networks at the systems biology level. Further defining the exosomal miRNA signaling in the RPE and the functional relations to microglial gene expression will expand our knowledge on better understanding the aging and degeneration processes of the RPE and retina.

## 4. Materials and Methods

### 4.1. Mice

Animal protocols were approved by the Institutional Animal Care and Use Committee (IACUC) of the University of Texas Medical Branch (UTMB) or the University of Oklahoma Health Sciences Center (OUHSC). B6129 mice (Stock number 101043) were obtained from Jackson Laboratory (Bar Harbor, ME, USA). Euthanasia was performed by exposing the animals to carbon dioxide delivered at 2 L/min through a three-stage gas regulator. All procedures were conducted in accordance with the ARVO Statement for the Use of Animals in Ophthalmic and Vision Research. Mice at age of 2 to 24 months were used for the study.

### 4.2. Purification of Exosomal RNA Released by Human RPE Cells or Mice RPE-Choroid-Sclera Explant and RNA-Seq Analyses

Human fetal RPE cells [30] were seeded on collagen-coated 100 mm dishes and cultured in alpha-modified Eagle’s medium supplemented with 10% fetal bovine serum (FBS) (Thermo Fisher Scientific, Waltham, MA, USA), 10 mL/L N1 supplements and 10 mL/L MEM non-essential amino acids (Sigma-Aldrich, St. Louis, MO, USA). Once cells reached confluence, the medium was changed to Dulbecco’s Modified Eagle Medium (DMEM) with 1 g/L glucose and 2% FBS [30]. At 1 and 4 weeks after postmitotic culture, cells were washed and changed with serum-free DMEM medium. After 16 h incubation, RPE-conditioned culture supernatant was collected. Extracellular vesicles (EVs) were isolated and their RNA was prepared from the conditioned media with an exoRNeasy kit (Qiagen, Germantown, MD, USA) following the manufacturer’s instructions. RNA quality assessment and sequencing were performed by BGI Genomics (San Jose, CA, USA).

For collecting EVs from mice RPE-choroid-sclera explant, eye globes were enucleated from euthanized 4–6 months (young) or 18–22 months (old) B6129 mice. After removing the anterior segment and retina, the posterior eye cups of RPE/choroid/sclera were cultured in serum-free DMEM medium for 16 h. Twelve eyes (6 mice) were used for one sample. The supernatant was collected and filtered through 0.45 mm filter. ExoRNeasy kit (Qiagen) was used to purify RNA from EVs.

Raw reads from RNA-seq were cleaned, mapped to human genome hg38 or mouse genome mm9, and counted using FeatureCounts [41]. Differential expression analyses between young and aged samples were performed using DeSeq2 [42].

### 4.3. Nanoparticle Tracking Analysis

The particle size and concentration of EVs in the culture medium conditioned by RPE-choroid-sclera explant were analyzed using an NanoSight NS300 system (Malvern PANalytical, Westborough, MA, USA), after 1:10 dilution in phosphate-buffered saline. Imaging video files were recorded and analyzed following the manufacturer’s instructions. Three repeated measurements were performed on each sample.

### 4.4. Western Blot Analyses of EVs

RPE/choroid/sclera explants were prepared as described above and cultured in serum-free DMEM medium for 16 h. EVs in the conditioned medium were isolated using an ExoQuick-TC kit (Systems Biosciences, Palo Alto, CA, USA), and lysed in buffer containing CelLytic™ M cell lysis reagent (Sigma-Aldrich) and 2× Laemmli Sample Buffer (Bio-Rad, Hercules, CA, USA) at 1:1 ratio, 10 mM glycerophosphate, 10 mM pyrophosphate, 1 mM NaF, 1 mM Na_3_VO_4_, and protease inhibitor cocktails. Samples were resolved on SDS-PAGE and transferred to nitrocellulose membranes (Bio-Rad). Membranes were probed with an antibody against TSG101 (Abcam, Cambridge, MA, USA), followed by AlexFluor 680-conjugated secondary antibody (Thermo Fisher). For detecting the immunoglobulin bands, only secondary IRDye 680RD donkey anti-mouse IgG antibody (LI-COR, Lincoln, NE, USA) was used. The signals were detected by an Odyssey Infrared Imaging System (LI-COR, Linclon, NE, USA) [30].

### 4.5. Quantitation of Cellular miRNAs

Mouse RPE/choroid tissue was harvested and lysed in TRIzol reagent (Thermo Fisher). Total RNA, including mRNA and miRNA, was purified with miRNeasy kit (Qiagen). MiRNAs were reverse-transcribed into cDNA using the Taqman microRNA reverse transcription kit (Thermo Fisher). The specific miRNA species were amplified and quantitated by q-RT PCR using predesigned Taqman™ MicroRNA assay (Thermo Fisher). For normalization, the expression levels of β-actin were measured in separate q-RT-PCR reactions using oligo dT primer (Promega, Madison, WI, USA) and Taqman probe-based assay [30]. Real-time PCR was performed on an ABS 7500 real-time PCR system (Applied Biosystems, Foster City, CA, USA). Results were presented as fold change, after being normalized to β-actin.

### 4.6. Fluorescent miRNA In Situ Hybridization (ISH)

In situ hybridization was performed with the ViewRNA ISH tissue assay kit (Thermo Fisher) following the manufacturer’s recommendations. The paraffin sections of posterior eyecups at 8 μm thickness were deparaffinized and digested with protease K at 40 °C for 15 minutes to unmask the RNA targets. Probes for miR-21 or scrambled control were hybridized with the samples for 2 h at 40 °C. Signals were further amplified with probe-specific amplifiers and label probes. Images were acquired on a Carl Zeiss AxioVision microscope equipped with ApoTome [43].

### 4.7. Culture of Mice Primary Retinal Microglia

Primary cultures of mouse retinal microglia were established from postnatal day 3 to 5 (P3 to P5) mice, following established methods with modifications [44,45,46]. Briefly, retina from 4–8 mice were collected and subjected to collagenase digestion at 70 unit/mL for 15 min at 37 °C. After filtration through 70 μm nylon strainer (Thermo Fischer), cells were collected by centrifugation at 300× *g* for 5 min. The pellet was resuspended in DMEM/F12 medium containing 20% FBS, 20% L929 cell-conditioned medium, 50 ng/mL granulocyte-macrophage colony stimulation factor (GM-CSF) [47] (Shenandoah Biotechnology), 2 mmol/L GlutaMAX (Thermo Fischer), 100 U/mL penicillin, and 100 µg/mL streptomycin, and seeded on either 6-well cell culture plates or transwell inserts for co-culture with the RPE.

The viability of cultured microglia was assessed with LIVE/DEAD Viability/Cytotoxicity kit (Thermo Fisher), using calcein AM and ethidium homodimer-1 to label live and dead cells, respectively (Figure S1A) [48]. Microglia routinely had >95% viability. Flow cytometry analyses were performed to further validate the surface markers of microglia (Figure S1B–D). Cells were treated with lipopolysaccharide (LPS) at 20 ng/mL for 16 h [49]. After blocking the FcγR with anti-CD16/32 antibody (Miltenyi Biotec, San Diego, CA, USA), cell surface antigens were labeled with the following antibodies: FITC anti-CD45 (Thermo Fisher), PE/Cyanine5 anti-CD11b (Thermo Fisher), PE/Cyanine5 anti-mouse CD40 (Thermo Fisher), Brillant Violet 605 anti-CX3CR1 (BioLegend), and Alexa Fluor647 anti-CCR2 (R&D Systems, Minneapolis, MN). Flow cytometry was performed on an Attune NxT flow cytometer (Thermo Fisher). Cultured microglia were CD45^+^, CD11b^+^, CX3CR1^+^, and CCR2⁻ [50]. The level of CD40 was markedly upregulated by LPS treatment (Figure S1D) [51,52].

### 4.8. Transfer of miRNA 21 from RPE to Microglia

Mouse RPE cell culture was established from B6129 mice at 2 to 3 months of age [53]. Cells used for imaging were grown on collagen (STEMCell Technologies, Cambridge, MA, USA)-coated cover slips. Cells at 50–60% confluency were transfected with FITC-conjugated miRNA21 mimic (Integrated DNA Technologies, Coralville, IA, USA) using Lipofectmine 2000 reagent (Thermo Fisher). Five h after transfection, the media with transfection reagent was removed. Cells were washed twice with phosphate-buffered saline and incubated in fresh medium for 16 h. The RPE-conditioned medium was then collected and used to treat cultured microglia to test the transfer of RPE-derived FITC-conjugated miRNA21. A co-staining of 100 nM LysoTracker Red (Thermo Fisher) for 40 min was performed to monitor the location of lysosomes. Live cell fluorescence imaging was performed, after nuclei staining with 4′,6-diamidino-2-phenylindole (DAPI) (Sigma–Aldrich).

### 4.9. Transfection of Microglia with miR-21 Mimics

Cultured mouse microglia at ~60% confluency on 6-well plates were incubated in Opti-MEM medium (Thermo Fisher) and transfected with miRIDIAN miR-21 mimics or negative control (Horizon Discovery, Lafayette, CO, USA) at 15 nM concentration, using Lipofactamine 2000 reagent. The miR-21 mimics were double-stranded RNA oligonucleotides based on mature miR-21-5p sequence. After 6 h, the transfection reagents were removed. Cells were cultured in fresh regular medium for 48 h, followed by treatment with nutulin or doxycycline (Sigma–Aldrich) at indicated concentrations for additional 16 h. RNA was isolated from microglia, and quantitative RT-PCR was performed to measure the mRNA expression of *p21*, *Cdc25a*, and *Daxx* [30].

## Supplementary Materials

The following are available online at https://www.mdpi.com/1422-0067/21/10/3541/s1.
