# Supplementary figures and images for: Exosomal MiRNA Transfer between Retinal Microglia and RPE
Source: Int J Mol Sci. 2020 May 17;21(10):3541. doi: 10.3390/ijms21103541 (PMC7279010; doi:10.3390/ijms21103541)

**A** calcein / ethidium homodimer

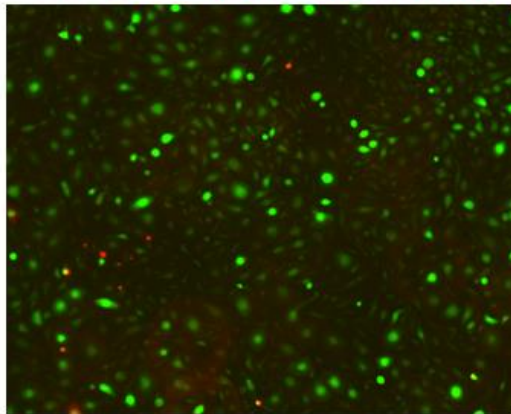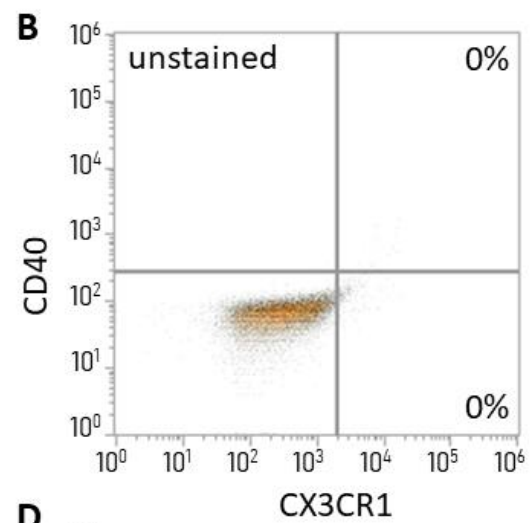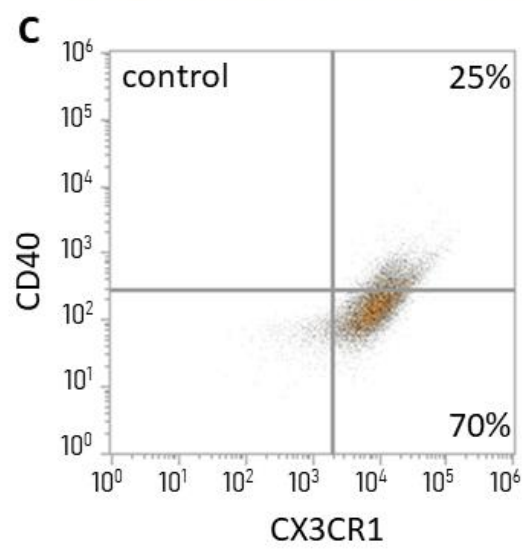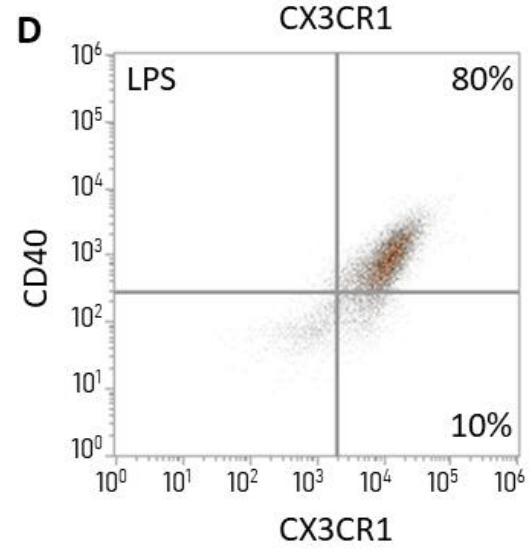

Supplement: Supplementary file 1 [file ijms-21-03541-s001.pdf]
